# Restricted cubic spline model analysis of the association between anal fistula and anorectal abscess incidence and body mass index

**DOI:** 10.3389/fsurg.2023.1329557

**Published:** 2024-01-08

**Authors:** Sangyu Ye, Zichen Huang, Lihua Zheng, Yuying Shi, Congcong Zhi, Ningyuan Liu, Yicheng Cheng

**Affiliations:** ^1^ Graduate School, Beijing University of Chinese Medicine, Beijing, China; ^2^ Proctology Department, China-Japan Friendship Hospital, Beijing, China

**Keywords:** anal fistula, anorectal abscess, BMI, overweight, obesity

## Abstract

**Objective:**

The epidemiological profile of anal fistula and anorectal abscess has not been well studied. Based on the results of a retrospective cross-sectional survey, we aimed to investigate the potential influential factors associated with anal fistula and anorectal abscess.

**Methods:**

We conducted a retrospective analysis of outpatients who visited the proctology department at China-Japan Friendship Hospital between January 2017 and May 2022. A comprehensive questionnaire was designed to collect potential influential factors, and according to formal anorectal examination and the corresponding diagnostic criteria, all the participants were divided into patients with anal fistula or perianal abscess and healthy control group. Multiple logistic regression was used to identify factors in significant association with anal fistula and perianal abscess. Additionally, we combined restricted cubic spline regression to examine the dose-response relationship between factors and the risk of developing anal fistula or anorectal abscess.

**Results:**

The present study included 1,223 participants, including 1,018 males and 206 females, with 275 anal fistulas, 184 anorectal abscesses, and 765 healthy controls. We found no statistically significant differences between patients and controls in basic information and preoperative assessment of life factors, except for body mass index. It was indicated that people with overweight or obesity were more prone to anal fistula (OR _overweight _= 1.35, 95% CI: 1.00–1.82, *P* = 0.047; OR _obesity _= 3.44, 95% CI: 2.26–5.26, *P* < 0.001) or anorectal abscess (OR _overweight _= 1.41, 95% CI: 1.00–1.99, *P* = 0.05; OR _obesity_: 2.24, 95% CI: 1.37–3.67, *P* = 0.001) than normal-weight individuals. The dose-response research indicated the J-shaped trend between the ascending BMI levels and the higher risk of suffering from anal fistula and anorectal abscess.

**Conclusions:**

Our findings indicate that overweight and obesity are risk factors for anal fistula and anorectal abscess, which plays a role in the prevention of anorectal diseases. This provides some theoretical basis for clinicians to provide health education to their patients.

## Introduction

Anal fistula and anorectal abscess are two challenging anorectal conditions. The attention they receive from specialists in this field is a testament to their difficulty to treat (1–3). An anal fistula is an infected channel that connects the skin outside the anus with the inside of the rectum, lined with glandular epithelial or granulation tissue. Common symptoms include lumps around the anus, hard nodules, recurring pain, redness, swelling and sometimes rupture (4). In the United States, approximately 20,000–25,000 people are affected by anal fistula each year (5), while in the United Kingdom, the total incidence rate of anal fistula is about 1.69 cases per 10,000 population annually (6). In clinical studies, males have a significantly higher incidence of anal fistula than females, with a ratio of 1.8:1 (7). Meanwhile, an anorectal abscess is a type of pyogenic infection that occurs when an anal gland becomes obstructed. The clinical symptoms include pain, fever, and the appearance of an anal bulge. This disease typically affects individuals between 20 and 40 years old and has a significant impact on patient quality of life. In the UK alone, approximately 18,000 patients are affected by anorectal abscesses each year (8), while in the United States this number ranges from 68,000 to 96,000 people annually (9). It's important to note that abscesses and fistulas are two phases of the same anorectal infection, with abscess being acute and fistula being chronic. If left untreated or mishandled during surgery, anorectal abscess can develop into anal fistula. Although neither condition is considered fatal, they can cause severe pain and discomfort for patients which negatively impacts their psychological state, which contains depression or anxiety (4). Therefore, it's crucial to take measures to prevent both anal fistulae and perianal abscesses.

Currently, the investigation of risk factors has proven to be advantageous in developing prevention and intervention strategies (10). Case-control studies have revealed that certain factors can increase the likelihood of anal fistula and anorectal abscess, including unhealthy diets, a history of diabetes, sedentary habits, lack of exercise, and irregular bowel movements (9, 11). Devaraj and Doctor Zheng et al. suggest that recent smoking is linked to the development of anal abscess or anal fistula. However, this influence diminishes after 5–10 years of smoking cessation (12, 13). M, Cioli V et al. highlight that emotional distress plays a role in the pathogenesis of anal fistula and emphasize the importance of psychological screening for patients with anorectal disorders (14). These specific dietary and lifestyle factors have been identified as risk factors for anal fistula and anorectal abscesses which can aid in constructing predictive models to prevent the occurrence of disease.

Moreover, obesity has reached epidemic proportions worldwide, accounting for more than 1.9 billion overweight and approximately 650 million obese adults. It is a recognized risk factor for the development of comorbid conditions such as cardiovascular disease, type 2 diabetes mellitus, malignancy, asthma, osteoarthritis, chronic back pain, obstructive sleep apnoea, non-alcoholic fatty liver disease, and gallbladder diseases (15). In clinical research, it has been discovered that individuals who are overweight or obese have a higher likelihood of developing anal fistulas or anorectal abscesses. However, there is currently no available information on whether obesity can be considered a predictive risk factor for these conditions. As such, our study sought to investigate the potential association between BMI and the prevalence of anal fistula and anorectal abscess.

## Methods

### Study subjects

Participants who visited the proctology department at China-Japan Friendship Hospital between January 2017 and May 2022 were included in the present study, including patients who seek medical treatment due to illness and a healthy control group for physical examination.

### Inclusion and exclusion criteria

The inclusion criteria of patients group: (1) Patients were diagnosed with anal fistula or anorectal abscess according to *Guidelines for the Treatment of anorectal abscesses and Fistulas*, which was developed by the American College of Colorectal Surgeons and the Parks Classification System in 2016 (9, 16). (2) Age equal to or greater than 18 years; (3) With no cellulitis, systemic signs of infection, or underlying immunosuppression; (4) With no serious cardiovascular, cerebrovascular, liver, kidney diseases, malignant tumor and hematopoietic system diseases; (5) Patients informed consent.

The exclusion criteria of patients group: (1) Atopic anal fistula due to tuberculosis, Crohn's disease, ulcerative colitis, etc.; (2) Combined with other anorectal diseases such as rectal polyps, rectal cancer, severe mixed hemorrhoids; (3) Females during pregnancy and lactation; (4) Allergic patients and psychiatric patients.

The healthy group consisted of participants with no severe anorectal diseases, except for mild cases of haemorrhoids and perianal skin disease.

### Basic characteristics and quality control

A comprehensive electronic questionnaire was designed for both anal fistula or anorectal abscess patients and healthy controls, which included information on socio-demographic, lifestyle characteristics, and medical history. Items from the questionnaire were selected *a priori* based upon published literature and clinical experience. In basic socio-demographic information, we concluded gender, age, job, family income, marital status, education, and body mass index (BMI), and family history. Body weight (to the nearest 0.1 kg) and height (to the nearest 0.1 cm) were measured by trained physicians. Lifestyle characteristics included eating patterns, smoking history, alcohol consumption, spicy/oily diet, and so on. Medical history contained the history of common anorectal disorders. Meanwhile, any history of hypertension, diabetes, and hyperlipidemia was recorded.

Notably, sedentary behavior refers to any waking behavior characterized by an energy expenditure ≤1.5 metabolic equivalents while in a sitting or reclining posture, which is defined by the Sedentary Behavior Research Network (9). In the present study, the sedentary mode was coded as ″0″ if sedentary time was less than 6 h per day, and as ″1″ if sedentary time was more than 6 h per day. Furthermore, dietary habits were evaluated by determining whether or not has a penchant for a spicy/oily diet. In the present study, the spicy/oily diet mode was coded as ″0″ if participants never eat spicy greasy food, and as ″1″ if participants eat spicy greasy food more than once a week.

BMI is calculated as weight (kg) divided by height (m) squared. Stages of BMI were classified into four stages based on the BMI values: skinny (BMI <18.5 kg/m^2^), normal (BMI = 18.5–24.0 kg/m^2^), overweight (BMI >24.0 kg/m^2^, BMI >28.0 kg/m^2^), and obese (BMI ≥28.0 kg/m^2^).

The doctors of anorectal department were responsible for explaining and circulating the questionnaires to all participants. Once the completed questionnaire was received, data from the electronic questionnaires were downloaded into Excel spreadsheets and cross-checked by trained staff to record demographic and clinical data on anal fistula and anorectal abscess from each patient's medical record. After excluding 68 invalid questionnaires with missing information, 1,223 qualified questionnaires were obtained within the specified timeframe.

### Ethical approval

This retrospective case-control study was approved by the Ethics Committee of China-Japan Friendship Hospital, and all patients included provided informed consent.

### Statistical analyses

Continuous variables that follow a normal distribution are presented as mean (standard deviation) or median (interquartile range). Categorical variables are presented as frequency (percentage). To compare the anal fistula group and non-anal fistula group, and anorectal abscess group and non-anal abscess group, chi-square test is used for categorical variables while *t*-test or Wilcoxon rank-sum test is used for continuous variables. Multiple logistic regression was employed to identify factors significantly associated with anal fistula and perianal abscess. According to different correction factors, we conducted three models. Model 1 identified uni-variate predictors (uncorrected model), whereas model 2 was corrected for age and sex. Model 3 was defined as a comprehensive model for adjusting other factors. Furthermore, we utilized restricted cubic spline regression to examine the dose-response relationship between factors and the risk of developing anal fistula or anorectal abscess. All statistical analyses were performed using STATA statistical software (version 14.0, Stata Corp MP). A two-sided *P* value < 0.05 was considered statistically significant.

## Results

### Basic demographics characteristics

Table 1 shows the baseline characteristics of all participants in the study. A total of 1,223 participants were included, with an average age of 37 years. The prevalence of anal fistula and anorectal abscesses was 22.5% and 15.0%, respectively. There were 1,018 males and 205 females, with 432 males having anal fistula or perianal abscess and only 27 females.

**Table 1:** Baseline characteristics of study participants in this study.

Characteristics	Anal fistula		*P*	Anorectal abscesses		*P*
	No (*n* = 948)	Yes (*n* = 275)		No (*n* = 1,039)	Yes (*n* = 184)	
Age, years	34.55 (29.5, 42.8)	34.4 (29, 42.3)	0.776	34.7 (29.5, 42.8)	34.4 (28.4, 42.2)	0.284
Gender			<0.001			0.005
Male	760 (80.2%)	258 (93.8%)		844 (81.2%)	174 (94.6%)	
Female	188 (19.8%)	17 (6.2%)		195 (18.8%)	10 (5.4%)	
Marital status			0.938			0.656
Married	593 (62.6%)	177 (64.4%)		651 (62.7%)	119 (64.7%)	
Unmarried	338 (35.7%)	94 (34.2%)		370 (35.6%)	62 (33.7%)	
Widowed	12 (1.3%)	3 (1.1%)		12 (1.2%)	3 (1.6%)	
Divorced	5 (0.5%)	1 (0.4%)		6 (0.6%)	0 (0%)	
Job			0.489			0.005
Student	21 (2.2%)	9 (3.3%)		25 (2.4%)	5 (2.7%)	
On-the-job	334 (35.2%)	98 (35.6%)		386 (37.2%)	46 (25%)	
Retired	26 (2.7%)	4.0 (1.5%)		28 (2.7%)	2 (1.1%)	
Other	567 (59.8%)	164 (59.6%)		600 (57.7%)	131 (71.2%)	
Drinking			0.579			0.152
Yes	218 (23.0%)	59 (21.5%)		228 (22%)	49 (26.8%)	
No	728 (77.0%)	216 (78.5%)		810 (78%)	134 (73.2%)	
Smoking			<0.001			0.737
Yes	51 (6.8%)	25 (15.6%)		67 (8.2%)	9 (9.2%)	
No	704 (93.2%)	135 (84.4%)		750 (91.8%)	89 (90.8%)	
Education			0.363			0.094
Junior secondary and below	71 (7.5%)	18 (6.5%)		75 (7.2%)	14 (7.6%)	
High school/secondary	256 (27.0%)	80 (29.1%)		299 (28.8%)	37 (20.1%)	
College/university	523 (55.2%)	140 (50.9%)		555 (53.4%)	108 (58.7%)	
Postgraduates and above	98 (10.3%)	37 (13.5%)		110 (10.6%)	25 (13.6%)	
BMI (kg/m^2^)	23.89 (21.93, 25.55)	24.77 (22.86, 26.89)	<0.001	24.00 (21.97, 25.93)	24.49 (22.62, 26.23)	0.005
Sedentary			0.118			0.664
Yes	553 (58.4%)	146 (53.1%)		597 (57.5%)	102 (55.7%)	
No	394 (41.6%)	129 (46.9%)		442 (42.5%)	81 (44.3%)	
Spicy and greasy			0.982			0.035
Yes	333 (35.2%)	97 (35.3%)		353 (34.0%)	77 (42.1%)	
No	613 (64.8%)	178 (64.7%)		685 (66.0%)	106 (57.9%)	
Other diseases			0.946			0.177
None	817 (86.2%)	239 (86.9%)		890 (85.7%)	166 (90.2%)	
Gastrointestinal diseases	123 (12.0%)	34 (12.4%)		141 (13.6%)	16 (8.7%)	
Non-gastrointestinal diseases	8 (0.8%)	2 (0.7%)		8 (0.8%)	2 (1.1%)	

BMI, body mass index.

### The risk factors of anal fistula and perianal abscess

The study results suggest that there was no normal distribution observed between the patients and control subjects in terms of basic information such as age, job, marital status, education, and preoperative life factors assessment including history of smoking, alcohol consumption, sedentary lifestyle and diet. However, statistically significant differences were found in BMI, gender and smoking habits between anal fistula patients and healthy individuals. As shown in Table 2, without considering other factors, the multivariate logistic regression model revealed that a lean body mass was a protective factor for the occurrence of anal fistula, with no statistically significant (Model 1: OR = 0.75, 95% CI: 0.29–1.99, *P* = 0.569; Model 2: OR = 0.98, 95% CI = 0.36–2.67, *P* = 0.975; Model 3: OR = 0.97, 95% CI = 0.29–3.18, *P* = 0.954). Inversely, overweight (Model 1: OR = 1.36, 95% CI: 1.01–1.83, *P* = 0.046; Model 2: OR = 1.16, 95% CI: 0.85–1.57, *P* = 0.349; Model 3: OR = 0.96, 95% CI: 0.64–1.44, *P* = 0.854) and obesity (Model 1: OR = 3.44, 95% CI: 2.26–5.26, *P* < 0.01; Model 2: OR = 3.16, 95% CI: 2.05–4.87, *P* < 0.01; Model 3: OR = 2.43, 95% CI: 1.38–4.29, *P* = 0.002) are the risk factors for anal fistula. Similarly, anorectal abscess patients showed significant differences compared to healthy controls in terms of BMI, job, education level and history of spicy/oily diet (*P* < 0.05). The multivariate logistic regression analysis indicated that a lean body mass was a protective factor for the occurrence of anal fistula, with no statistically significant (Model 1: OR = 0.93, 95% CI: 0.32–2.72, *P* = 0.894). However, in Model 2 and Model 3, the lean body mass seems to be risk factor for anal fistula (Model 2: OR = 1.22, 95% CI: 0.41–3.68, *P* = 0.718; Model 3: OR = 1.70, 95% CI = 0.46–6.30, *P* = 0.424. Overweight (Model 1: OR = 1.41, 95% CI: 1.00–2.00, *P* = 0.049; Model 2: OR = 1.22, 95% CI: 0.86–1.74, *P* = 0.271; Model 3: OR = 1.32, 95% CI: 0.81–2.14, *P* = 0.271) and obesity (Model 1: OR = 2.24, 95% CI: 1.37–3.67, *P* = 0.001; Model 2: OR = 2.04, 95% CI: 1.23–3.36, *P* = 0.005; Model 3: OR = 2.96; 95% CI: 1.52–5.76, *P* = 0.001).

**Table 2:** Univariate and multivariate logistic regression analysis of the association between anal fistula and anorectal abscess incidence and body mass index.

		Anal fistula				Anorectal abscesses			
Model	BMI grouping	OR	*z*	*P*	95% CI	OR	*z*	*P*	95% CI
Model 1	Overly thin	0.75	−0.57	0.569	0.29–1.99	0.93	−0.13	0.894	0.32–2.72
	Normal				Ref.				Ref.
	Overweight	1.36	2.00	0.046	1.01–1.83	1.41	1.97	0.049	1.00–2.00
	Obesity	3.44	5.73	<0.001	2.26–5.26	2.24	3.20	0.001	1.37–3.67
Model 2	Overly thin	0.98	−0.03	0.975	0.36–2.67	1.22	0.36	0.718	0.41–3.68
	Normal				Ref.				Ref.
	Overweight	1.16	0.94	0.349	0.85–1.57	1.22	1.10	0.271	0.86–1.74
	Obesity	3.16	5.22	<0.001	2.05–4.87	2.04	2.78	0.005	1.23–3.36
Model 3	Overly thin	0.97	−0.06	0.954	0.29–3.18	1.70	0.80	0.424	0.46–6.30
	Normal				Ref.				Ref.
	Overweight	0.96	−0.18	0.854	0.64–1.44	1.32	1.10	0.271	0.81–2.14
	Obesity	2.43	3.08	0.002	1.38–4.29	2.96	3.20	0.001	1.52–5.76

Model 2, adjusted for age and gender; Model 3, adjusted for age, gender, job, marital status, education, smoking, drinking, sedentary, spicy and greasy.

### Dose-response analyze

**Figure 1:**
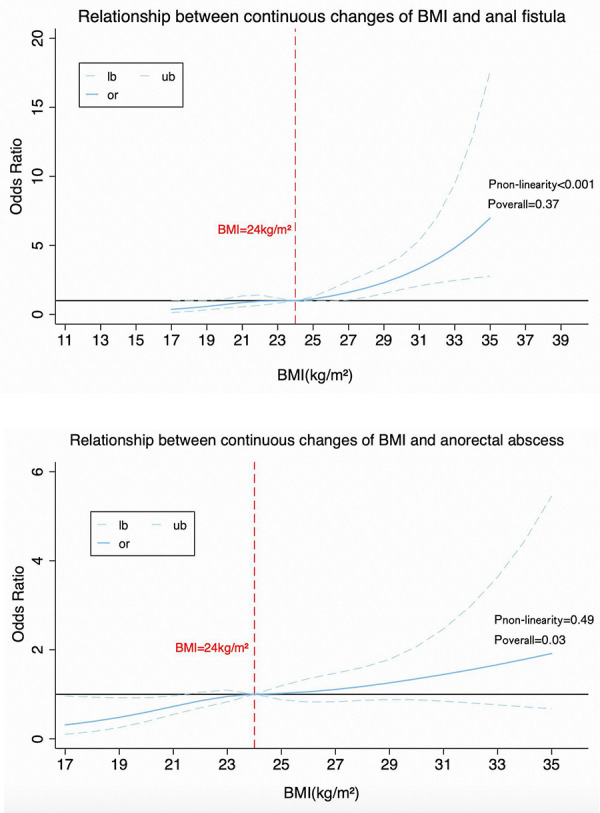
Dose-response curves of anal fistula and anorectal abscess risk associated with continuously changing BMI.

**Figure 2:**
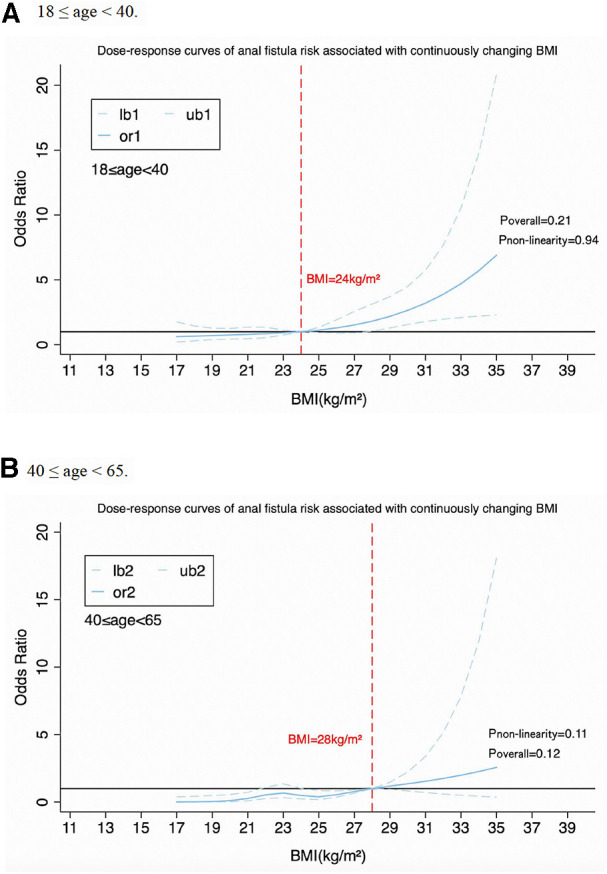
Association between BMI and anal fistula in different age groups. Panel A shows people aged 18–40, Panel B shows people aged 40-65.

By using Restricted Cubic Spline Regression (17), a dose-response relationship between continuous changes in BMI and incidence of anal fistula (P_overall_ < 0.001, P_non−linearity_ = 0.37) and anorectal abscess (P_overall_ = 0.03, P_non−linearity_ = 0.49) has been found (see Figure 1). A “J” characteristic curve means the risk of anal fistula increases progressively with rising BMI. We also found the relative BMI cut points across different age groups. In detail, between the ages of 18–40, when BMI is greater than 24 kg/m^2^, the risk of developing anal fistula increases. While between the ages of 40 and 65, if BMI exceeds 28 kg/m^2^, there is a clear increasing trend in the risk of developing anal fistula (see Figure 2). Although P_non−linearity_ and P_overall_ were no statistical significance, there were Figure 2 showing an upward trend of the relationship between BMI and anal fistula.

## Discussion

To our knowledge, the association between BMI and anal fistula or anorectal abscess remains controversial (18–20). This study aimed to investigate the relationship between BMI and the incidence of anal fistula and anorectal abscess. We found that overweight and obese people are more prone to anal fistula or anorectal abscess than normal-weight individuals. The dose-response research indicated a J-shaped trend between increasing BMI levels and a higher risk of suffering from anal fistula and anorectal abscess. Importantly, the effect of BMI on anal fistula and anorectal abscess varied by age. Accordingly, the implication of this study is a call for action to pay attention to obesity control in patients with anal diseases around the world.

As economies rapidly develop, obesity has emerged as a significant health risk factor due to changes in people's lifestyles and dietary habits. This has led to an increase in cardiovascular diseases, endocrine diseases, malignant tumors, and osteoarticular system diseases, which cause nearly 3 million deaths annually worldwide (21). In addition, obesity is a risk factor for postoperative complications such as SSI and sepsis after colorectal surgery (22). These complications have been associated with negative economic impact, increased morbidity, extended postoperative hospital stay, readmission, and death (23). In developed countries, the prevalence of overweight and obesity among men increased from 28.8% in 1980 to 36.9% in 2013, for women, it rose from 29.8% to 38%. Over the same period, a slower but still notable increase was observed for children, adolescents, and adults in developing countries (from 8.1% to 12.9%) (24).

Our findings appear to be physiologically and pathologically plausible. The association between overweight or obesity and anal fistula and anorectal abscess may involve the following mechanisms. On the one hand, overweight or obesity is associated with abnormalities in lipid and glucose metabolism (25, 26). High glucose and lipid levels have predisposed to anal sinus and anal gland infections, which can lead to anorectal abscesses and fistulae due to abnormalities in cell-mediated immune and phagocytic functions associated with poor glycemic control (27). On the other hand, the patient's blood glucose rises and the glycogen content of the skin tissue increases, creating good conditions for bacterial infection, as if forming a bacterial “petri dish” (28). After gland infection, pus is transferred from the anal gland to the space between the sphincters, forming an abscess that leads to the formation of a fistula. An anorectal abscess containing intestinal flora usually transforms into an anal fistula at a later stage. The incidence of post-abscess fistulae is almost 33% (27, 29, 30), however, when studied accurately, internal fistula tracts can be found in almost 80% of cases (31). Clinically, we also found that obese people have the characteristics less mobile and sedentary. Once the body's immunity declines, the defective bactericidal activity of neutrophils increases the risk of bacterial infections in patients with abnormal glucose and lipid metabolism (32), and anorectal abscess or anal fistula will follow. Except for the mechanisms mentioned above, gender, smoking, emotion, sedentary habits, unhealthy diets, and other aspects may modify the effects of BMI on anal fistula or anorectal abscess (9, 11–14). Almost all the included studies have adjusted the confounding factors, and obesity remained to be a significant risk factor, indicating the consistency of the findings.

Anal fistula and anorectal abscess are thorny anorectal diseases, and postoperative uncomfortable brings great trouble to patients. At present, the principle of treatment for anal fistula is the complete removal of the internal orifice and its associated fistula tissue while preserving the function of the anal sphincter as much as possible, such as fistula treatment by fistulotomy, LIFT, and internal anal advancement flap (16). The principle of treatment for anorectal abscess is drainage and patency. The current surgical treatment is also the mainstay, such as Needle aspiration treatment (33), incision and seton drainage, and etc. (34). Nevertheless, postoperative pain, urinary incontinence, anal incontinence, and healing time of the wound after anal fistula and anorectal abscesses should not be underestimated. During the preliminary healing process, patients can irritate the wound during defecation, causing pain. García-Botello et al. found that the severity of urinary incontinence increased with the complexity of the fistula (35). Dong et al. showed that damage to the postoperative sphincter can lead to anal incontinence (36). Moreover, postoperative healing time for anal fistulas and anorectal abscesses is also a long process, requiring at least 3 months to recover (37). In conclusion, prevention and early intervention are particularly crucial for overweight and obese individuals who are at high risk of developing these diseases.

The study has several limitations that need to be acknowledged. Firstly, as a single-center study, the results may not be as applicable to other centers as they would be in a multicenter study. Secondly, BMI standards differ across various regions of the world and Asia; therefore, further research is required to establish links between BMI and anal fistula or anorectal abscess. Thirdly, it is necessary to assess the local imaging features of all enrolled patients, such as fat thickness and other relevant factors. Fourthly, BMI may reflect the obesity characteristics of hyperglycemia, hyperlipidemia and systemic immune dysfunction in some patients. However, it is important to distinguish that the underlying mechanism between these diseases and anal fistula and anorectal abscess may not be related to BMI. Last, since this was a retrospective study, there could have been other factors that were not considered during statistical analysis which might have influenced the outcomes. It is recommended that more well-designed multicenter studies should be conducted to explore the relationship between preoperative BMI and anal fistula or anorectal abscess with different baseline indices.

## Conclusions

In summary, the restricted cubic spline model suggests that being overweight or obese increases the risk of developing anal fistula and anorectal abscess, highlighting their role in preventing anorectal diseases. However, further well-designed studies conducted across multiple centers are necessary to explore the correlation between preoperative BMI and anal fistula or anorectal abscess with varying baseline indices.

## Data Availability

The original contributions presented in the study are included in the article/Supplementary Material, further inquiries can be directed to the corresponding author.
